# Efficacy and follow‐up of humanized anti‐BCMA CAR‐T cell therapy in relapsed/refractory multiple myeloma patients with extramedullary‐extraosseous, extramedullary‐bone related, and without extramedullary disease

**DOI:** 10.1002/hon.2958

**Published:** 2022-01-10

**Authors:** Wei Li, Meijing Liu, Ting Yuan, Lixiang Yan, Rui Cui, Qi Deng

**Affiliations:** ^1^ Department of Hematology The First Teaching Hospital of Tianjin University of Traditional Chinese Medicine Tianjin China; ^2^ Department of Hematology Tianjin First Central Hospital School of Medicine Nankai University Tianjin China

**Keywords:** anti‐BCMA CAR‐T, efficacy, extramedullary multiple myeloma, follow‐up, refractory, relapsed

## Abstract

The prognosis of patients with multiple myeloma (MM) with extramedullary disease (EMD) remains poor. A high overall response rate (ORR) has been reported following anti‐B‐cell maturation antigen (BCMA) chimeric antigen receptor (CAR)‐T cell therapy in relapsed/refractory (R/R) patients with MM; however, data on patients with EMD remain limited. Herein, we compared and analyzed the efficacy and long‐term follow‐up of anti‐BCMA CAR‐T cell therapy in R/R MM patients with extramedullary‐extraosseous (EM‐E), extramedullary‐bone related (EM‐B), and without extramedullary disease. No difference in the ORR was observed between the three groups. The long‐term efficacy of anti‐BCMA CAR‐T cell therapy in the EM‐E group was worse than that in patients without EMD and with EM‐B. In the EM‐E group, disease progression was the reappearance of extramedullary lesions without an increase in the MM cell percentage or M protein level. Although no difference in the proportion of CAR‐T cells was detected among the three groups, the EM‐E group might exhibit a relatively high grade of cytokine release syndrome following anti‐BCMA CAR‐T therapy. Interleukin‐6 levels in the without EMD group were lower than those in the EM‐E and EM‐B groups. However, given the small number of cases in the three groups, statistical analysis was not performed.(ChiCTR1800017051 and ChiCTR2000033925).

## 1 NTRODUCTION

Multiple myeloma (MM) is a hematologic malignancy characterized by proliferative disorders of neoplastic plasma cells. Although proteasome inhibitors, monoclonal antibodies, immunomodulatory agents, and autologous hematopoietic stem cell transplantation (auto‐HSCT) could significantly improve efficacy and survival,^1^, ^2^, ^3^ some patients develop relapsed/refractory (R/R) MM with an extremely poor prognosis.^4^, ^5^, ^6^ MM cells are often not confined to bone marrow, migrating from the bone marrow, infiltrating into various extramedullary organs throughout the body, and even infiltrating the peripheral blood.^7^ This extramedullary MM (EMM) is a rare type of MM, with the lesion termed extramedullary disease (EMD).^8^ It might develop at initial diagnosis, during relapse, or follow‐up. Two types of EMDs are known to exist: extramedullary‐extraosseous (EM‐E), leading to soft tissue tumors at a site far from the bone, and extramedullary‐bone related (EM‐B), which invades the surrounding soft tissues.^9^, ^10^ Although several new EMD treatments are available, the prognosis of MM patients with EMD remains poor.^11^, ^12^, ^13^ Anti‐B cell maturation antigen (anti‐BCMA) is a member of the tumor necrosis factor receptor superfamily, mainly expressed in MM cells of almost all patients.^14^ Therefore, anti‐BCMA is considered an ideal target for chimeric antigen receptor (CAR)‐T cell therapy in patients with R/R MM.^15^, ^16^ A high overall response rate (ORR) has been reported following anti‐BCMA CAR‐T cell therapy in patients with R/R MM^17^, ^18^; however, data on MM patients with EMD are limited. Herein, we compared and analyzed the efficacy, safety, and long‐term follow‐up of humanized anti‐BCMA CAR‐T cell therapy in R/R MM patients with EM‐E, EM‐B, and without EMD.

## 2 PATIENTS AND METHODS

### 2.1 Patients enrolled in the study

Twenty‐one patients were diagnosed with R/R MM and enrolled in clinical trials of humanized anti‐BCMA CAR‐T cell therapy *(ChiCTR1800017051 and ChiCTR2000033925)* between September 2018 and June 2020, including six patients with at least one EM‐E, six patients with at least one EM‐B, and nine patients without any assessable EMD. All patients had BCMA expression in MM cells at enrollment. The cutoff date was 31 July 2021. All patients were observed after anti‐BCMA CAR‐T cell therapy for more than 12 months unless death occurred due to R/R MM.

### 2.2 Preparation of humanized anti‐BCMA CAR‐T cells and anti‐BCMA CAR‐T cell therapy

Peripheral blood mononuclear cells were collected from patients with R/R MM and isolated by Ficoll density gradient centrifugation. CD3+ T cells were selected using CD3 microbeads (Miltenyi Biotec, Inc.). CD3+ T cells (5 × 10^6^) were transduced with lentiviral supernatant from 293T cells transfected with humanized anti‐BCMA CAR plasmid (20 μg, lenti‐BCMA‐2rd‐CAR; Shanghai Genbase Biotechnology Co. Ltd.). On day 12–15 cultivation, transduction efficiencies of anti‐BCMA CAR were analyzed by flow cytometry (FCM) (BD Biosciences).

Before anti‐BCMA CAR‐T cell infusion, all patients with R/R MM received lymphodepleting chemotherapy with fludarabine (30 mg/m^2^) and cyclophosphamide (400 mg/m^2^) from day 4 to day 2. All patients received autologous humanized anti‐BCMA CAR‐T cells (2 × 10^6^ cells/kg) on day 0. No patient received auto‐HSCT after anti‐BCMA CAR‐T cell therapy.

### 2.3 Criteria for diagnosis and evaluation criteria for therapeutic efficacy

Diagnoses of R/R MM and EM‐E and EM‐B and clinical response to the humanized anti‐BCMA CAR‐T cell therapy were assessed according to the International Myeloma Working Group Guidelines uniform response criteria for MM.^19^ Herein, the proportion of MM cells was determined using bone marrow morphology and flow cytometric analysis. M protein levels were detected using immunofixation electrophoresis. EM‐E and EM‐B were detected using computed‐tomography, positron emission tomography, and magnetic resonance imaging. MM cells in the pleural effusion were detected using FCM.

The therapeutic efficacy of all patients with R/R MM was evaluated monthly after anti‐BCMA CAR‐T cell infusion in the first three months; then, efficacy was evaluated every three months. The follow‐up was performed from the date of humanized anti‐BCMA CAR‐T cell infusion until patient death or study cutoff date. Clinical responses included stringent complete response (sCR), complete response (CR), very good partial response (VGPR), partial response (PR), minimal response, stable disease (SD), and progressive disease. ORR (sCR, CR, VGPR, and PR), overall survival (OS), and progression‐free survival (PFS) were also assessed.

### 2.4 Adverse events following humanized anti‐BCMA CAR‐T cell therapy

We evaluated Adverse events (AEs) associated with humanized anti‐BCMA CAR‐T cell therapy. The grade of cytokine release syndrome (CRS) was determined according to the National Cancer Institute Common Terminology Criteria for Adverse Events v4.03.^20^ Immune effector cell‐associated neurotoxicity syndrome (ICANS) was used to assess neurotoxicity.^21^

FCM was used to assess the proportion of anti‐BCMA CAR‐T cells in peripheral blood on days 0, 4, 7, 14, 28, and 60 after infusion; the proportion of these cells was also determined in pleural effusion. Cytokine levels, including interleukin‐6 (IL‐6), IL‐2R, and tumor necrosis factor‐α (TNF‐α), were assessed on days 0, 7, 14, 28, and 60 using enzyme‐linked immunosorbent assay.

### 2.5 Statistical analysis

Data are expressed as the mean ± standard error (SE). Probabilities of PFS and OS were estimated using the Kaplan‐Meier method and compared using the log‐rank test. All statistical analyses were performed using GraphPad Prism 7 and SPSS 17.0. Statistical significance was set at *p* < 0.05.

## 3 RESULTS

### 3.1 Characteristics of patients with R/R MM

Table 1 lists the characteristics of patients with R/R MM enrolled in our study. At enrollment, all patients with *R/R* MM showed BCMA expression in MM cells, determined by FCM.

**TABLE 1:** Baseline characteristics of the R/R multiple myeloma patients

	Pt	Sex (F/M)	Age	KPS	Subtype	ISS Stage	Extramedullary disease	Time of extramedullary disease from diagnosis (months)	High‐risk Cytogenetics	Numbers of Prior therapy	Murine BCMA CAR‐T cell therapy before	Auto‐HSCT or radiotherapy before
EM‐E	**1**	M	57	90	*k* light chain	III	EM‐B/EM‐E	5	*t*(4;14), *t*(14;16), Del(17p)	14	No	HSCT (1 time)
	**2**	F	69	80	IgG‐λ	III	EM‐E	5	*t*(4;14)	9	No	No
	**3**	M	59	100	*k* light chain	III	EM‐B/EM‐E	4	*t*(14;16)	8	Yes	Radiotherapy
	**4**	F	53	90	IgG‐κ	II	EM‐B/EM‐E	5	*t*(14;16)	9	No	Radiotherapy
	**5**	F	38	90	*k* light chain	II	EM‐B/EM‐E	6	None	17	No	HSCT (2 times)
	**6**	F	55	80	IgG‐λ	III	EM‐E	5	Del(17p), *t*(14;16)	14	No	No
EM‐B	**1**	F	52	90	IgG‐λ	III	EM‐B	6	*t*(14;16)	14	No	HSCT (2 times)
	**2**	F	55	90	IgG‐κ	II	EM‐B	3	Del(17p), t(4;14)	10	No	Radiotherapy
	**3**	F	71	80	IgD‐λ	III	EM‐B	4	Del(17p)	17	No	Radiotherapy
	**4**	M	45	100	*k* light chain	II	EM‐E	9	None	7	No	HSCT (2 times)
	**5**	M	61	90	*k* light chain	II	EM‐B	7	*t*(14;16)	9	No	HSCT (1 time)
	**6**	F	67	80	IgG‐λ	III	EM‐E	5	Del(17p), *t*(4;14)	13	No	No
EMD**‐**	**1**	F	69	80	IgG‐λ	II	‐	‐	*t*(14,16)	13	No	No
	**2**	M	58	100	*k* light chain	III	‐	‐	*t*(4;14)	11	No	No
	**3**	M	63	80	IgG‐λ	III	‐	‐	*t*(14;16)	5	No	HSCT (1 time)
	**4**	M	66	90	IgA‐κ	II	‐	‐	*t*(14;16), Del(17p)	16	No	No
	**5**	F	55	90	IgG‐λ	II	‐	‐	None	7	No	No
	**6**	F	52	90	IgA‐λ	II	‐	‐	Del(17p)	10	No	HSCT (1 time)
	**7**	F	72	80	IgG‐κ	III	‐	‐	*t*(14;16)	8	No	No
	**8**	F	77	80	IgG‐κ	III	‐	‐	None	13	No	No
	**9**	F	42	100	IgG‐κ	III	‐	‐	*t*(14;16)	5	No	No

Abbreviations: BCMA, anti‐B‐cell maturation antigen; EM‐B, Extramedullary‐bone related; EMD‐, Without extramedullary disease group; EM‐E, Extramedullary‐extraosseous; HSCT, hematopoietic stem cell transplantation; ISS, International Staging System; KPS, Karnofsky Performance Scale.

### 3.2 Transduction and amplification efficiency, infusion dose of humanized anti‐BCMA CAR‐T cells

The mean humanized anti‐BCMA CAR transduction efficiency for final products of the EM‐E, EM‐B, and without EMD group were 37.87 ± 9.65%, 36.32 ± 8.04%, and 39.57 ± 9.08%, respectively. On harvesting anti‐BCMA CAR‐T cells, the mean number of cells for the three groups was (4.05 ± 1.82 × 10^6^), (3.68 ± 1.24 × 10^6^), and (4.37 ± 0.81 × 10^6^) cells/kg. On day 0, all groups received a dose of (2.18 ± 0.41 × 10^6^), (2.08 ± 0.19 × 10^6^), and (2.05 ± 0.15 × 10^6^) cells/kg autologous humanized anti‐BCMA CAR‐T cell infusion.

### 3.3 Clinical response to humanized anti‐BCMA CAR‐T therapy

**FIGURE 1:**
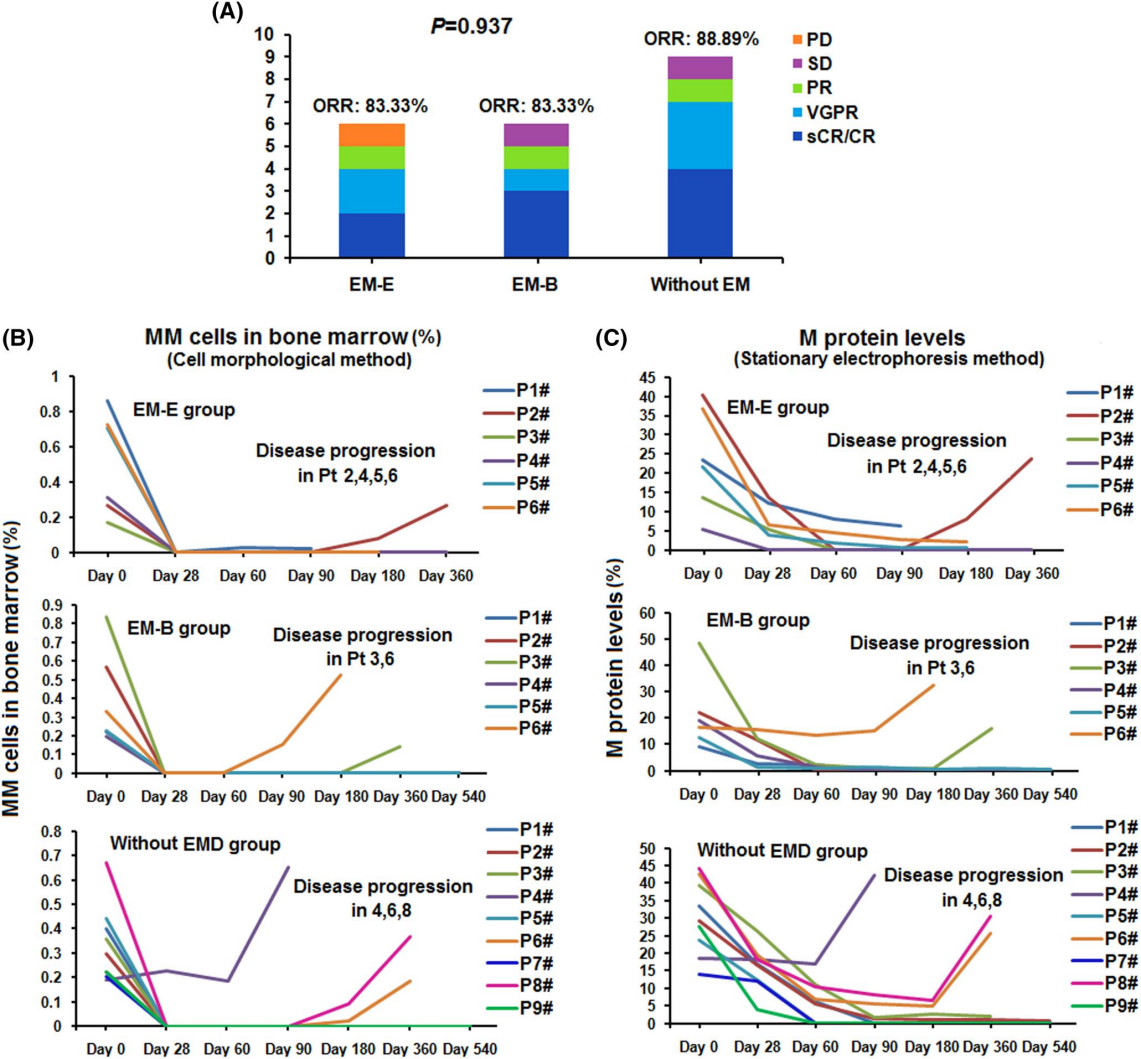
Clinical responses of the humanized anti‐BCMA CAR‐T cell therapy. (A) The clinical response to humanized anti‐BCMA CAR T therapy. The overall response rate (ORR) in Extramedullary‐extraosseous (EM‐E), Extramedullary‐bone related (EM‐B) and without extramedullary (EM) group was 83.33%, 83.33%, and 88.89% respectively. (B) The multiple myeloma (MM) cell percentages in bone marrow were observed after the anti‐BCMA CAR T cell infusion. (C) The M protein levels in the peripheral blood were observed after the anti‐BCMA CAR T cell infusion

We evaluated the efficacy of all 21 patients with R/R MM every month after anti‐BCMA CAR‐T cell infusion. The median time to reach optimal response was 2.5 months (1–4 months), 2.8 months (2–4 months), and 2.3 months (1–4 months), while ORRs were 83.33%, 83.33%, and 88.89% in EM‐E, EM‐B, and without EMD groups, respectively (Figure 1A). No difference in ORR was noted between the three groups (*p* = 0.937).

MM cell percentages in the bone marrow and M protein levels in peripheral blood were determined after anti‐BCMA CAR‐T cell infusion (Figure 1B,C). Except for patient 4 in the without EMD group (Pt_without_ 4), who achieved SD following anti‐BCMA CAR‐T cell therapy, the MM cell percentages in the bone marrow and M protein levels in peripheral blood decreased to different degrees in all patients with R/R MM anti‐BCMA CAR‐T cell infusion. In all patients with R/R MM who reached ORR, no MM cells were detected in the bone marrow, and the M protein level decreased to varying degrees. MM cells in the bone marrow and M protein levels in two patients who reached PD and SD in the EM‐E and EM‐B groups exhibited the same characteristics as patients who reached ORR. However, the MM cell percentage and M protein level of the Pt_without_ 4 who reached SD failed to decline after anti‐BCMA CAR‐T cell therapy.

In EM and EB groups, assessable EMD of patients with R/R MM who reached ORR disappeared or shrunk on reaching optimal response. However, it failed to shrink in patients who did not achieve the ORR.

### 3.4 Disease re‐progression after anti‐BCMA CAR‐T cell therapy

On disease re‐progression after anti‐BCMA CAR‐T cell therapy, we assessed the proportion of bone marrow MM cells and M protein level in peripheral blood. In the EM‐E group, the proportion of MM cells and M protein level increased again in patient 2 (Pt_EM‐E_ 2) when EMD recurred. However, no increase was detected in the proportion of MM cells and M protein level in Pt_EM‐E_ 1, 4, 5, and 6 following EMD recurrence. In these patients, disease progression was noted as the reappearance of EM lesions (both primary and new sites). In the EM‐B group, the proportion of MM cells and M protein level increased again with EMD recurrence in patient 3 (Pt_EM‐B_ 3) and Pt_EM‐B_ 6. In the control group, the proportion of MM cells and M protein level increased again in Pt_without_ 4, 6, and 8 following disease re‐progression (Figure 1B and C).

In EM‐E and EM‐B groups, all patients with R/R MM who attained the ORR showed the original EMD volume enlargement or the emergence of new EMD following disease re‐progression. In bone marrow MM cells, anti‐BCMA expression was analyzed by FCM following disease re‐progression. Anti‐BCMA expression in detectable bone marrow MM cells was positive or weakly positive in these patients.

### 3.5 Survival time following humanized anti‐BCMA CAR‐T therapy

**FIGURE 2:**
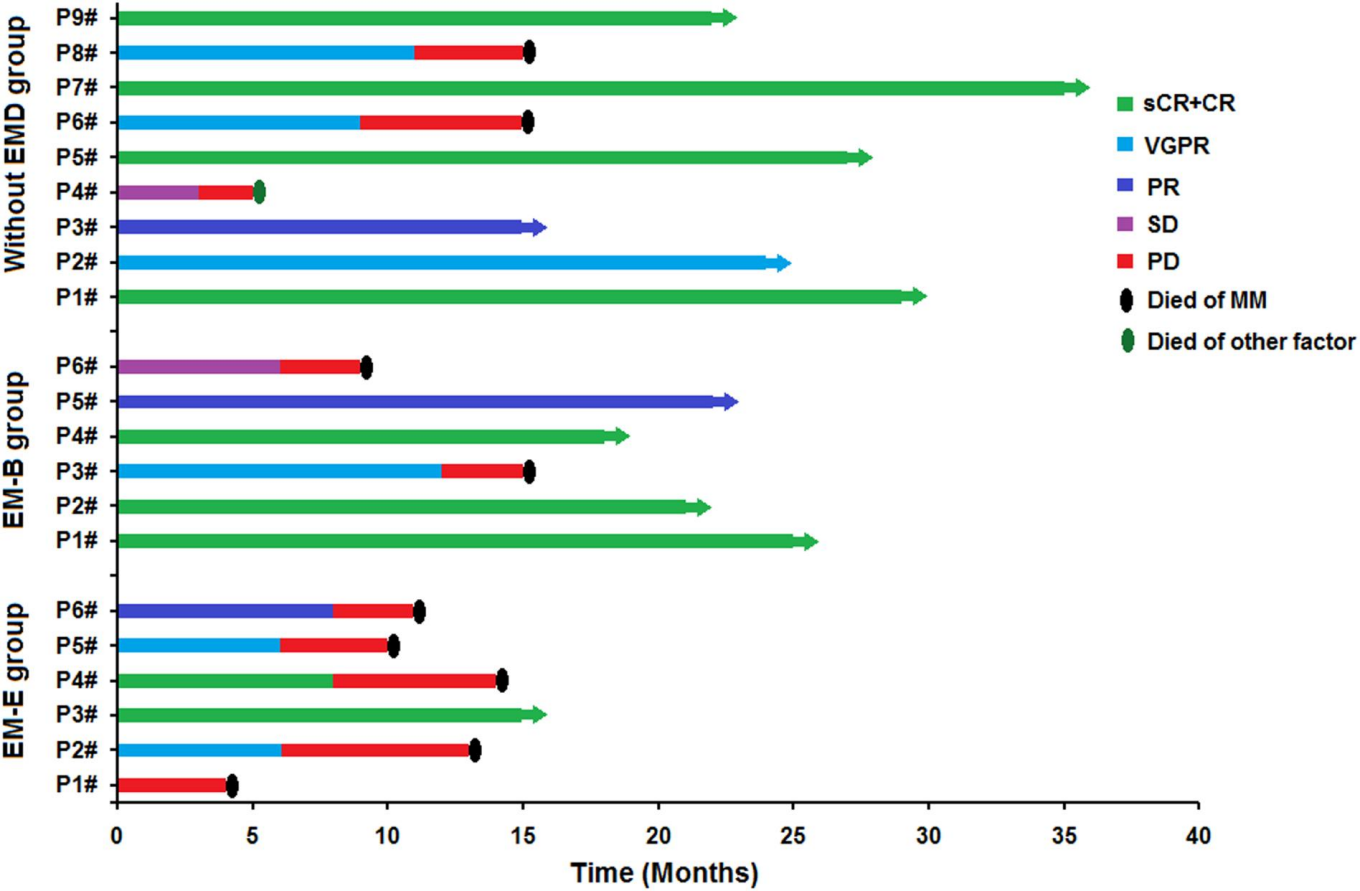
The survival observation of the humanized anti‐BCMA CAR‐T cell therapy

During follow‐up after the optimal response time, four patients in the EM‐E group and two in the EM‐B group showed disease progression and died due to primary disease. Although three patients in the without EMD group showed disease progression, two died owing to the primary disease; the other patient died of other factors (cerebrovascular disease) (Figure 2).

PFS rates in patients with R/R MM were 50%, 83.33%, and 88.89% in the EM‐E, EM‐B, and without EMD groups, respectively, after 180 days of CAR‐T cell infusion; after 360 days, PFS rates were 16.67%, 66.67%, and 66.67%, respectively. OS rates in the patients with R/R MM were 83.33%, 100%, and 88.89% in the EM‐E, EM‐B, and without EMD groups, respectively, after 180 days of CAR‐T cell infusion; after 360 days, OS rates were 33.33%, 83.33%, and 88.89%, respectively. Given the small number of cases in the three groups, statistical analysis was not performed.

### 3.6 Proportions of humanized anti‐BCMA CAR‐T cells in peripheral blood

**FIGURE 3:**
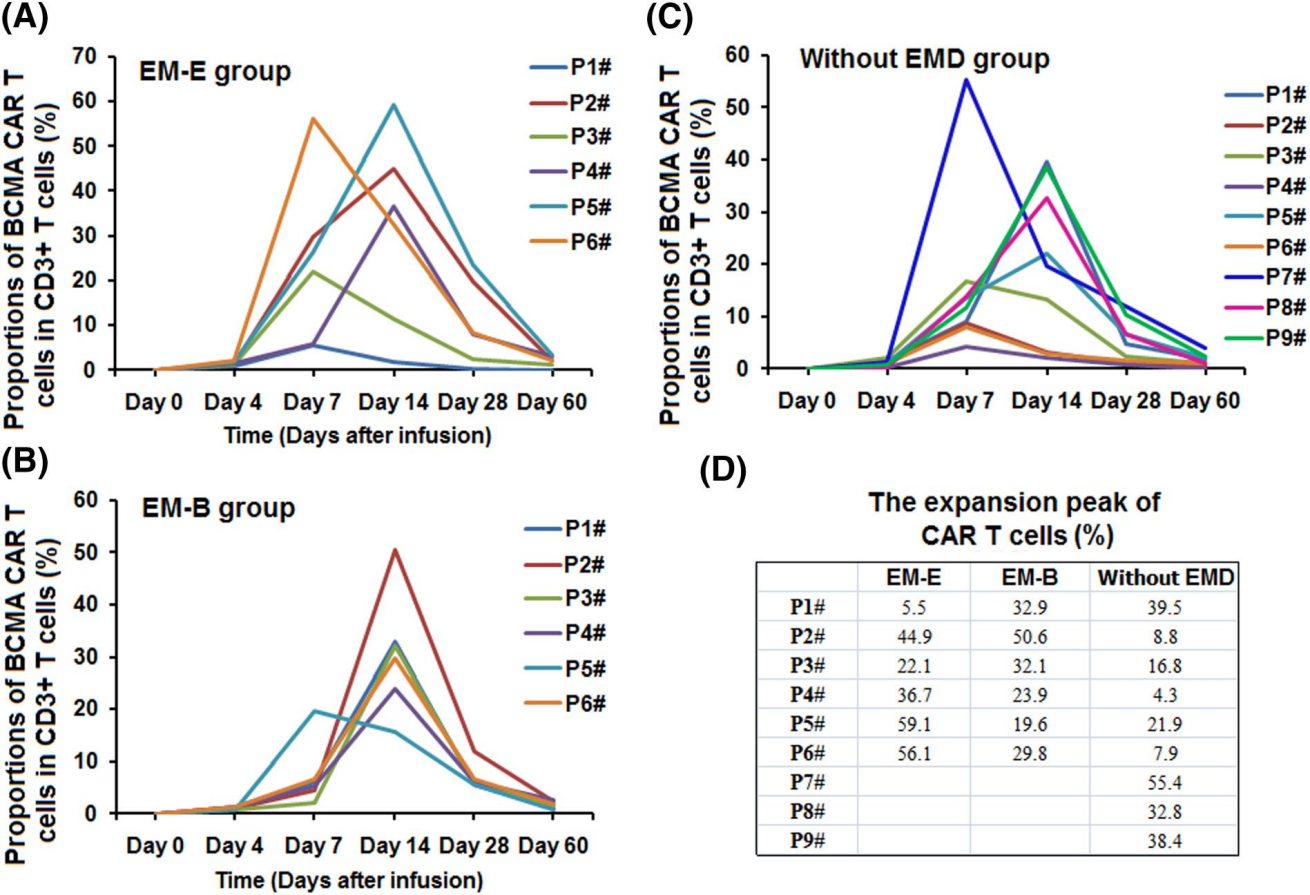
The proportions of the anti‐BCMA CAR‐T cells in the therapy. (A–C) The proportions of humanized anti‐BCMA CAR T cells in peripheral blood in the three groups. (D) There was no difference in the expansion peaks of the CAR‐T cells in the three groups

The proportion of anti‐BCMA CAR‐T cells was detected at days 0, 4, 7, 14, 28, and 60 by FCM after CAR‐T cell infusion (Figure 3A–C). For CD3+ T cells in peripheral blood, the median expansion peak of the anti‐BCMA CAR‐T cells was 37.4 (interquartile range [IQR] 25.8, 53.3)% in the EM‐E group, 31.5 (IQR 25.4, 32.7)% in the EM‐B group, and 25.9 (IQR 8.8–38.4)% in the EM‐E group. Expansion peaks of humanized anti‐BCMA CAR‐T cells in the three groups are shown in Figure 3D.

### 3.7 Safety and adverse effects

CRS and ICANS grades in the three groups receiving anti‐BCMA‐CAR‐T cell therapy is listed in Table 2. No patient died from CRS or ICANS during therapy. Grade 3 and 4 CRS and grade 2 ICANS were detected in the EM‐E and EM‐B groups, respectively.

**TABLE 2:** The grades of CRS and ICANS in anti‐BCMA‐CAR T cell therapy

	EM‐E		EM‐B		Without EMD	
	CRS	ICANS	CRS	ICANS	CRS	ICANS
P1#	1	0	1	0	2	0
P2#	3	2	2	0	1	0
P3#	2	1	4	2	1	0
P4#	2	0	1	0	1	0
P5#	3	0	2	0	1	0
P6#	4	2	3	0	1	0
P7#					2	0
P8#					2	1
P9#					2	0

Abbreviations: CRS, cytokine release syndrome; EM‐E, Extramedullary‐extraosseous; EM‐B, Extramedullary‐bone related, EMD‐, Without extramedullary disease group; ICANS, Immune effector cell‐associated neurotoxicity syndrome.

**FIGURE 4:**
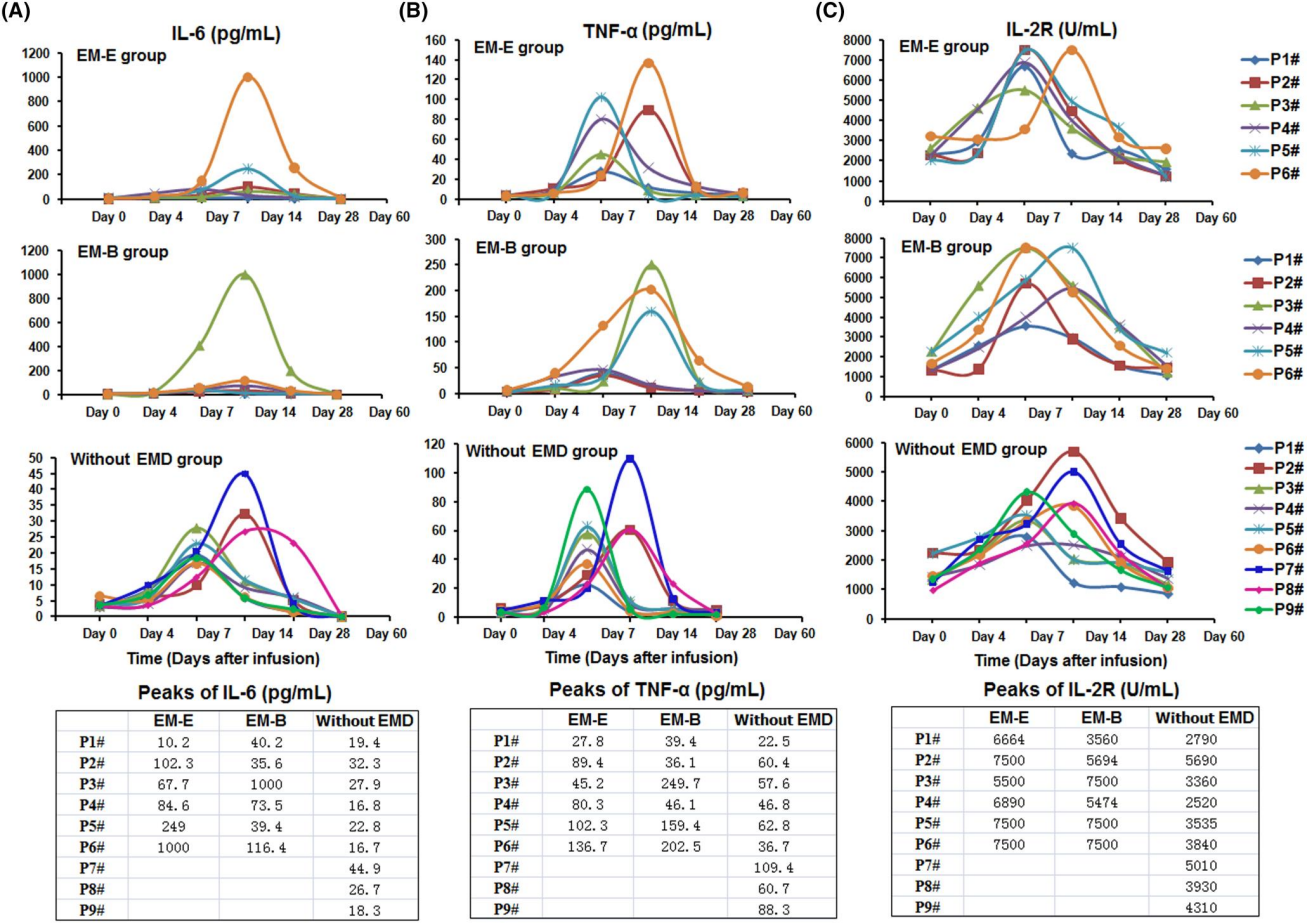
The serum level of IL‐6, TNF‐α and IL‐2R

IL‐6, TNF‐α, and IL‐2R levels peaked 4–7 days after anti‐BCMA CAR‐T cell infusion. Peaks of IL‐6, TNF‐α, and IL‐2R are shown in Figure 4A–C. Herein, IL‐6 peaks were markedly high in most patients in the EM‐E and EM‐B groups. IL‐2R peaks in patients with R/R MM in the without EMD group were lower than those in EM‐E and EM‐B groups. Given the small number of cases in the three groups, statistical analysis was not performed.

Following anti‐BCMA CAR‐T cell therapy, patients developed fever with or without chills, fatigue, edema, headache, nausea, tachycardia, and other symptoms 4–8 days after infusion. These symptoms recovered 16–42 days after CAR‐T cell infusion. Hematologic toxicity was deemed grade 1–4 in the anti‐BCMA CAR‐T cell therapy, occurring from 7 to 12 days and recovered 18–60 days after CAR‐T cell infusion (Table 3). No patient died from secondary infections due to hematologic toxicity.

**TABLE 3:** Adverse events in the three groups

	EM‐E group (*n* = 6)	EM‐B group (*n* = 6)	Without EMD group (*n* = 9)
Coagulopathy	2 (33.3%)	2 (33.3%)	2 (22.2%)
Gastrointestinal	4 (66.7%)	3 (50.0%)	2 (22.2%)
Creatinine increased			
Grade 0–2	4 (66.7%)	4 (66.7%)	7 (77.8%)
Grade ≥ 3	2 (33.3%)	2 (33.3%)	2 (22.2%)
Transaminase increases			
Grade 0–2	3 (50.0%)	3 (50.0%)	7 (77.8%)
Grade ≥ 3	3 (50.0%)	3 (50.0%)	2 (22.2%)
Cardiopulmonary			
Grade 0–2	4 (66.7%)	5 (83.3%)	8 (88.9%)
Grade ≥ 3	2 (33.3%)	1 (16.7%)	1 (11.1%)
Hematological toxicity			
Leukopenia			
Grade 0–2	4 (66.7%)	4 (66.7%)	6 (66.7%)
Grade ≥ 3	2 (33.3%)	2 (33.3%)	3 (33.3%)
Anemia			
Grade 0–2	4 (66.7%)	4 (66.7%)	6 (66.7%)
Grade ≥ 3	2 (33.3%)	2 (33.3%)	3 (33.3%)
Thrombocytopenia			
Grade 0–2	3 (50.0%)	3 (50.0%)	5 (55.6%)
Grade ≥ 3	3 (50.0%)	3 (50.0%)	4 (44.4%)

Abbreviations: EM‐B, Extramedullary‐bone related; EMD‐, Without extramedullary disease group; EM‐E, Extramedullary‐extraosseous.

The patients received methylprednisolone, antipyretic drugs, and symptomatic therapy to overcome their AEs. Patients in the EM‐E and EM‐B groups diagnosed with grade 3 and 4 CRS received tocilizumab during therapy. In addition, patients in the EM‐E and EM‐B groups diagnosed with grade 2 ICANS received dexamethasone to overcome serious AEs. No patient in the control group received tocilizumab or dexamethasone in our study.

## 4 DISCUSSION

EMD is defined as the proliferation of malignant MM cells outside the confines of bone marrow. In addition, EMD should exclude plasma cell leukemia and solitary plasmacytomas.^8^ It should be noted that EMD can develop at the time of initial diagnosis (15% incidence), at relapse, or during follow‐up (20% incidence).^22^, ^23^ However, data are limited in terms of incidence, prevalence, clinical characteristics, laboratory characteristics, and response to new drugs for EMD.^24^, ^25^, ^26^ Currently, no guidelines are available for the treatment of MM patients with EMM. In previous reports, the OS of patients with EMM did not typically exceed 3 years.^27^ In a recent retrospective multi‐institutional study, autologous stem cell transplant showed a survival benefit for both EM‐E and EM‐B.^28^ However, the prognosis of MM patients with EMD is poor, even with HSCT, and survival typically does not exceed 3 years.^29^, ^30^ In particular, the median OS of patients with relapse after auto‐HSCT was less than 1 year.^31^ Some studies have suggested that following the use of proteasome inhibitors and/or immunomodulatory agents, the PFS and OS of patients with EMM were less pronounced than those in MM patients without EMD.^32^, ^33^ In general, the prognosis for EM‐E is worse than EM‐B.^34^

Anti‐BCMA CAR‐T cell therapy showed satisfactory efficacy and prolonged the survival time of patients with R/R MM in previous clinical trials. The ORR of anti‐BCMA CAR‐T cell therapy was 85%, with 45% of patients reaching CR.^18^, ^35^ What is the efficacy of anti‐BCMA CAR‐T cells in the treatment of patients with R/R MM with EMD? The PFS and OS of R/R MM patients with EMD were lower than those of R/R MM patients without EMD.^36^, ^37^ However, clinical data are limited, except for a few case reports or a subset of EMD patients in clinical trials.^38^, ^39^, ^40^, ^41^ The efficacy and safety of anti‐BCMA CAR‐T cells in the two types of R/R MM patients with EMD (EM‐E and EM‐B) have been rarely reported.

Herein, we compared and analyzed the efficacy and follow‐up of humanized anti‐BCMA CAR‐T cell therapy in R/R MM patients with EM‐E, EM‐B, and without EMD. First, we observed no difference in ORRs between the EM‐E, EM‐B, and without EMD groups on evaluating the efficacy of all 21 patients with R/R MM. However, we noted differences in survival time between the EM‐E, EM‐B, and without EMD groups after humanized anti‐BCMA CAR‐T therapy. In the three groups, the PFS and OS rates in patients with R/R MM were similar at 180 days after CAR‐T cell infusion. However, the patients with R/R MM in the EM‐E group had no preponderant PFS and OS rates at 360 days after CAR‐T cell infusion. Given the small number of cases in the three groups, statistical analysis was not performed. Accordingly, we need to expand the number of cases for further investigation. Therefore, the long‐term efficacy of anti‐BCMA CAR‐T cell therapy in R/R MM patients with EM‐E remains unsatisfactory. R/R MM patients with EM‐E should receive maintenance therapy such as auto‐HSCT, radiotherapy, and immunomodulatory agents as early as possible prior to disease re‐progression. To maintain satisfactory outcomes following anti‐BCMA CAR‐T therapy. In order to maintain the efficacy of CAR‐T therapy for longer, we need to explore the methods of further therapy after anti‐BCMA CAR‐T therapy in R/R MM patients with EM‐E.

In case of disease re‐progression after anti‐BCMA CAR‐T therapy, the MM cell percentages in the bone marrow and M protein levels in peripheral blood of R/R MM patients with EM‐E are not necessarily consistent with further disease progression. In some patients, particularly in the EM‐E group, disease progression was simply a reappearance of EM lesions (both at primary and new sites).

The grades of CRS and ICANS in R/R MM patients without EMD were lower than those in the EM‐E and EM‐B groups. These results suggest that patients in the EM‐E and EM‐B groups might exhibit a relatively high grade of CRS and ICANS following anti‐BCMA CAR‐T therapy, which should be considered when determining their therapeutic course. In addition, serum IL‐6 levels in patients without EMD were lower than those in the other two groups, indicating that attention should be paid to potential side effects in R/R MM patients in the EM‐E and EM‐B groups during anti‐BCMA CAR‐T cell therapy. Assessing a larger sample size could provide more definitive results.

In conclusion, the long‐term efficacy of anti‐BCMA CAR‐T cell therapy in R/R MM patients with EM‐E was considerably worse than in patients without EMD and those with EM‐B. Therefore, further therapy after anti‐BCMA CAR‐T cell therapy to maintain the efficacy of CAR‐T therapy for longer is necessary for this patient group, possibly prolonging survival. In addition, attention should be paid to potential side effects in R/R MM patients with EM‐E.

## CONFLICT OF INTEREST

All authors have no conflict of interest to report.

## ETHICS STATEMENT

This study was approved by the Medical Ethics Committee of the Department of Hematology, Tianjin First Center Hospital (Tianjin, China). (Approved No. of ethic committee: 2015002X and 2020N028KY). The Clinical trial in our study was registered at http://www.chictr.org.cn/index.aspx as ***ChiCTR1800017051*** and ***ChiCTR2000033925***. The patient gave their written informed consent in accordance with the Declaration of Helsinki. The patients agreed to have their data used in our study.

## AUTHOR CONTRIBUTION

Concept and design: Qi Deng. Drafted or revised the manuscript: Wei Li, Meijing Liu, Ting Yuan and Rui Cui. Acquisition of data: Wei Li and Lixiang Yan. Analysis and interpretation of data: Wei Li. Writing, review and/or revision of manuscript: Wei Li. Study supervision: Qi Deng.

### PEER REVIEW

The peer review history for this article is available at https://publons.com/publon/10.1002/hon.2958.

## Data Availability

All data generated or analyzed during our study are included in this published article.
